# Cardiovascular Risks With SGLT2 Inhibitors in Clinical Practice Among Patients With Type 2 Diabetes

**DOI:** 10.1001/jamanetworkopen.2024.41765

**Published:** 2024-10-30

**Authors:** Hsuan-Yu Su, Chen-Yi Yang, Yu-Hsuan Lee, Pei-Fang Su, Yi-Chia Liu, Huang-Tz Ou

**Affiliations:** 1 Institute of Clinical Pharmacy and Pharmaceutical Sciences, College of Medicine, National Cheng Kung University, Tainan, Taiwan; 2 Department of Statistics, National Cheng Kung University, Tainan, Taiwan; 3 Clinical Innovation and Research Center, National Cheng Kung University Hospital, Tainan, Taiwan; 4 Department of Pharmacy, College of Medicine, National Cheng Kung University, Tainan, Taiwan

## Abstract

**Importance:**

Cardiovascular disease (CVD) can be recurrent during type 2 diabetes (T2D) progression in this aging population. The effectiveness of sodium-glucose cotransporter 2 inhibitor (SGLT2i) therapy on total (ie, first and subsequent) CVD among patients with T2D in clinical practice remains uncertain.

**Objective:**

To analyze the comparative association of SGLT2i vs dipeptidyl peptidase 4 inhibitor (DPP4i) therapy with total CVD among patients with T2D in clinical practice.

**Design, Setting, and Participants:**

This retrospective cohort study used electronic medical records at the National Cheng Kung University Hospital, a leading medical center in Taiwan, from 2015 through 2021. Adult patients with T2D who initiated first use of the study drugs from 2016 through 2019, with up to 6 years of follow-up, were identified.

**Main Outcomes and Measures:**

The primary outcomes included total composite CVD events and individual CVD subtypes (ie, atrial fibrillation, coronary heart disease, heart failure, stroke, myocardial infarction, and transient ischemic attack). A shared frailty model analysis was used to assess the association of treatment with repeat CVD events. Data from patients at high risk for CVD recurrence were further analyzed. Data were analyzed from September 1, 2022, to December 31, 2023.

**Results:**

Overall, 8384 patients with T2D were identified (mean [SD] age, 63.7 [12.4] years; 4645 [55.4%] male). A total of 1632 propensity score–matched pairs of SGLT2i (mean [SD] age, 57.8 [12.0] years; 673 [41.2%] female and 959 [58.8%] male) and DPP4i (mean [SD] age, 58.2 [12.9] years; 655 [40.1%] female and 977 [59.9%] male) users were included. SGLT2i was associated with reduced total CVD risk vs DPP4i therapy (hazard ratio [HR], 0.82 [95% CI, 0.69-0.98]) but not the first CVD event (with the use of SGLT2i therapy were more prominent for patients at high risk of CVD (ie, HR, 0.70 [95% CI, 0.62-0.80] for individuals with estimated glomerular filtration rate lower than 60 mL/min/1.73 m^2^; HR, 0.70 [95% CI, 0.64-0.78]; for individuals having any diabetes-related complications; and HR, 0.72 [95% CI, 0.65-0.80] for individuals with a history of CVD) compared with the overall cohort. Among patients at high risk of CVD, greater reduced total CVD burden associated with SGLT2i therapy was observed for women vs men (eg, HR, 0.59 [95% CI, 0.49-0.72] in the subgroup with CVD history).

**Conclusions and Relevance:**

In this cohort study of patients with T2D, the use of SGLT2is vs DPP4is was associated with reduced total cardiovascular burden, suggesting that long-term use of this therapy may optimize treatment benefit among patients with chronic CVD. The SGLT2i-associated benefit among patients with high risk of CVD encourages the prioritization of SGLT2i use for these vulnerable individuals.

## Key Points

**Question:**

Is the use of sodium-glucose cotransporter 2 inhibitors (SGLT2is) vs dipeptidyl peptidase 4 inhibitors (DPP4is) associated with reduced total cardiovascular disease (CVD) risks for patients with type 2 diabetes (T2D)?

**Findings:**

In this cohort study of 1632 propensity score–matched pairs with T2D, the use of SGLT2is vs DPP4is was associated with an 18% decrease in total CVD risks for the overall cohort. High risk for CVD recurrence was associated with the greatest benefit of treatment (up to a 30% reduction in risk).

**Meaning:**

Findings from this cohort study suggest that long-term use of SGLT2i therapy should be encouraged, especially for patients at high risk for CVD recurrence, to alleviate excess CVD burden.

## Teaser

This cohort study compares the effectiveness associated with sodium-glucose cotransporter 2 inhibitor vs dipeptidyl peptidase 4 inhibitor therapies on total cardiovascular disease risk and risk of atrial fibrillation, coronary heart disease, heart failure, stroke, myocardial infarction, and transient ischemic attack among patients with type 2 diabetes.

## Introduction

Approximately one-third of patients with type 2 diabetes (T2D) are affected by cardiovascular disease (CVD), including coronary artery disease, heart failure (HF), and stroke,^1,2^ which impose enormous health and economic burdens on individuals and health care systems.^2,3^ Given their progressive nature, CVD can be recurrent or exacerbated by an established sequence of cardiovascular events, aging, poor glycemic control, and impaired kidney function (eg, the presence of chronic kidney disease [CKD]).^4^ Hence, it is essential to consider repeated CVD events in addition to the first event when determining the total burden attributed to CVD in patients with T2D.^5^

Sodium-glucose cotransporter 2 inhibitors (SGLT2is), a relatively new class of glucose-lowering agents (GLAs) with cardiovascular benefits,^6,7,8^ are recommended to patients with T2D and established CVD or at high risk of CVD.^9,10,11^ To fully assess the SGLT2i-associated cardioprotective effect, several post hoc analyses of cardiovascular outcome trials have been conducted, with the first and recurrent hospitalization for HF and composite cardiovascular events following SGLT2i therapy measured.^12,13,14,15^ However, caution should be taken when interpreting these results given the limited follow-up time (eg, 4.2 years),^15^ selective trial populations, lack of active comparator, and only specific cardiovascular events of interest (eg, hospitalization for HF).^13^

Against this backdrop, the present study assessed data from a longitudinal cohort of patients in a clinical setting who had T2D to determine the effectiveness of SGLT2i therapy compared with another commonly prescribed class of second-line GLAs, namely, dipeptidyl peptidase 4 inhibitors (DPP4is), for total cardiovascular conditions. These cardiovascular conditions included both the first and recurrent events after treatment initiation and a broad spectrum of cardiovascular subtypes (ie, atrial fibrillation [AF], coronary heart disease [CHD], HF, hemorrhagic and ischemic strokes, myocardial infarction [MI], and transient ischemic attack). Our analyses used an advanced shared frailty model that can account for the dependence of event recurrence within individuals.

## Methods

### Data Source

This retrospective cohort study used electronic medical records (EMRs) from National Cheng Kung University Hospital (NCKUH) in Taiwan from 2015 to 2021. NCKUH is the leading medical center in southern Taiwan, with an average of 6310 outpatient visits and 141 inpatient visits per day.^16^ This study was approved by the Institutional Review Board of NCKUH, which waived the requirement for obtaining informed consent because of the retrospective analysis of anonymous data. The reporting of this study followed the Strengthening the Reporting of Observational Studies in Epidemiology (STROBE) reporting guideline for cohort studies.

### Cohort Identification

Individuals 18 years or older diagnosed with T2D (defined as having 2 outpatient visits with a T2D diagnosis [*International Classification of Diseases, Ninth Revision, Clinical Modification* (*ICD-9-CM*) codes 250.x0, 250.x2, where x = 0 to 9; or *ICD-10-CM* code E11] within a year or 1 outpatient visit with T2D diagnosis and any prescription for GLAs within the same year) and who initiated treatment with SGLT2is or DPP4is from January 1, 2016, though December 31, 2019, were identified from NCKUH’s EMRs. Only stable users were included for analyses. The index date was defined as the first date of DPP4i or SGLT2i prescription, and each patient was followed up from the index date to the end of 2021. Propensity score matching (PSM) on a series of clinically significant patient characteristics—demographics (eg, age, sex), comorbidities (estimated using the Charlson Comorbidity Index), and laboratory data (eg, glycated hemoglobin [HbA_1c_])—was performed to enhance the between-treatment group comparability at baseline (eMethods, eTable 1, and eTable 2 in Supplement 1). Patients with missing data in the matching covariates were further excluded from analysis. The eFigure in Supplement 1 provides the flowchart for patient selection.

### Study Outcomes

The study outcome of interest was a composite of nonfatal CVD events, which included AF, CHD, HF, hemorrhagic and ischemic strokes, MI, and transient ischemic attack. The occurrence of cardiovascular events was measured using *ICD-9-CM* and *ICD-10-CM* codes (eTable 3 in Supplement 1).

### Statistical Analysis

First, analyses of clinical outcomes that are well known to be positively associated with SGLT2i therapy were conducted to examine the internal validity of the study procedures.^17^ That is, given apparent SGLT2i-associated cardiovascular benefits (especially for the time to first CVD event),^6,7,8^ a Cox proportional hazards regression model analysis was performed to estimate the comparative risk of SGLT2i vs DPP4i therapy for the first CVD event (as a positive control outcome) following treatment initiation.^17^ Next, a shared frailty model analysis was conducted to examine the comparative effectiveness of SGLT2i vs DPP4i in reducing the risk of total CVD events. This analysis was designed to account for the dependence among recurrent CVD events within individuals and the unmeasured heterogeneity attributable to various baseline clinical characteristics that could not be explained by the observed covariates across individuals.^18^ For example, patients with a greater number of recurrent CVD events may have higher CVD risks than those with a smaller number of events. The joint frailty approach using frailty terms (ie, random effect), therefore, accounts for the heterogeneous risk for time to subsequent event between individuals. The Cox proportional hazards regression model, which includes only the first event, assumes an independent correlation between subsequent events, which may not accurately estimate the overall disease burden of interest (ie, CVD).

Subgroup analyses were further conducted for patients having (1) poor kidney function, with estimated glomerular filtration rate (eGFR) lower than 60 mL/min/1.73m^2^, (2) multiple diabetic complications, as measured by an adapted Diabetes Complications Severity Index (aDCSI) score higher than 0 (range, 0-13 with higher scores indicating greater severity), and (3) established CVD within 1 year before the index date (history of CVD). All of the aforementioned analyses (ie, PSM, Cox proportional hazards model, and shared frailty model) were assessed again within each subgroup. Moreover, to test whether the association of the treatment with the outcome was modified by patient baseline characteristics, the interactions terms of treatment exposure (ie, SGLT2is vs DPP4is) with (1) age at the index date, (2) sex, and (3) number of oral GLAs prescribed in the year before the index date were separately included in the shared frailty model analysis. All data analyses were performed using SAS software, version 9.4 (SAS Institute Inc). A 2-sided value of *P* < .05 was considered statistically significant.

## Results

**Figure 1.**
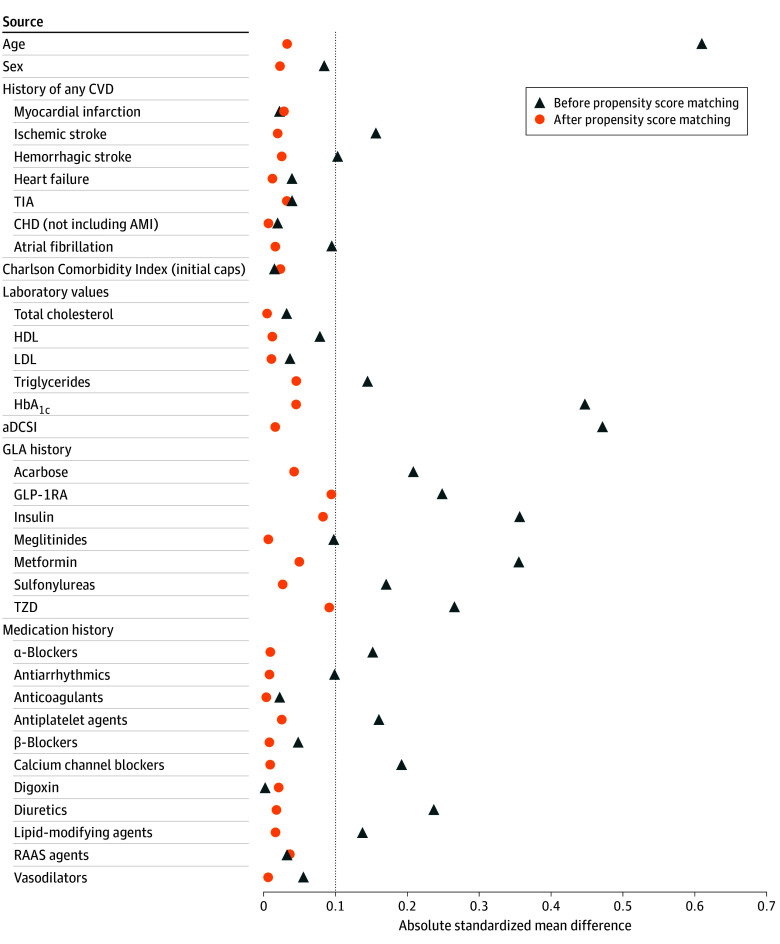
Absolute Standardized Mean Differences of Baseline Characteristics Between SGLT2i and DPP4i Groups Before and After Propensity Score Matching in the Overall Study Cohort Baseline characteristics were measured in the year before and at the index date (ie, the date of newly stable SGLT2i or DPP4i use in the study period). History of any cardiovascular disease (CVD) was determined from inpatient, outpatient, or emergency department medical records. A standardized mean difference greater than 0.1 indicates a significant between-group difference in the patient baseline characteristic. aDCSI indicates adapted Diabetes Complications Severity Index; AMI, acute myocardial infarction; CHD, coronary heart disease; DPP4is, dipeptidyl peptidase 4 inhibitors; GLA, glucose-lowering agent; GLP-1RA, glucagon-like peptide-1 receptor agonist; HbA_1c_, glycated hemoglobin; HDL, high-density lipoprotein; LDL, low-density lipoprotein; RAAS, renin-angiotensin-aldosterone system; SGLT2is, sodium-glucose cotransporter 2 inhibitors; TIA, transient ischemic attack; and TZD, thiazolidinedione.

From 2016 through 2019, 8384 patients with T2D were identified (mean [SD] age, 63.7 [12.4] years; 3739 [44.6%] female and 4645 [55.4%] male): 1632 with newly stable SGLT2i use and 6752 with newly stable DPP4i use (eTable 1 and eTable 2 in Supplement 1). After PSM, 1632 matched pairs of SGLT2i (mean [SD] age, 57.8 [12.0] years; 673 [41.2%] female and 959 [58.8%] male; mean [SD] HbA_1c_, 8.6% [1.4%]; to convert to proportion of total hemoglobin, multiply by 0.01]) and DPP4i (eg, mean [SD] age, 58.2 [12.9] years; 655 [40.1%] female and 977 [59.9%] male; mean [SD] HbA_1c_, 8.5% [1.8%]) users were obtained for analyses, with a greater level of between-group comparability in terms of baseline characteristics (Figure 1). The mean (SD) follow-up time of the overall study cohort ranged between 2.9 (1.9) years (ie, from the index date to the first cardiovascular event) and 4.8 (1.0) years (ie, from the index date until the end of follow-up) (eTable 1 in Supplement 1).

In the matched pairs of SGLT2i and DPP4i users, there were 585 in the group with eGFR lower than 60 mL/min/1.73m^2^, 1062 in the group with aDCSI scores higher than 0, and 458 in the group having established CVD (eTable 4 in Supplement 1). Compared with the overall study cohort, these subgroups generally had slightly higher percentages of patients with prior CVD at baseline. That is, the event rate of CHD in the overall cohort ranged from 21.0% to 21.2% compared with 27.9% to 28.7% for patients with an eGFR lower than 60 mL/min/1.73m^2^, 32.5% to 34.1% for patients with aDCSI scores higher than 0, and 74.5% to 75.5% for patients having established CVD. Female patients accounted for approximately 70% of cases in the subgroups of eGFR lower than 60 mL/min/1.73m^2^ and having a history of CVD.

**Figure 2.**
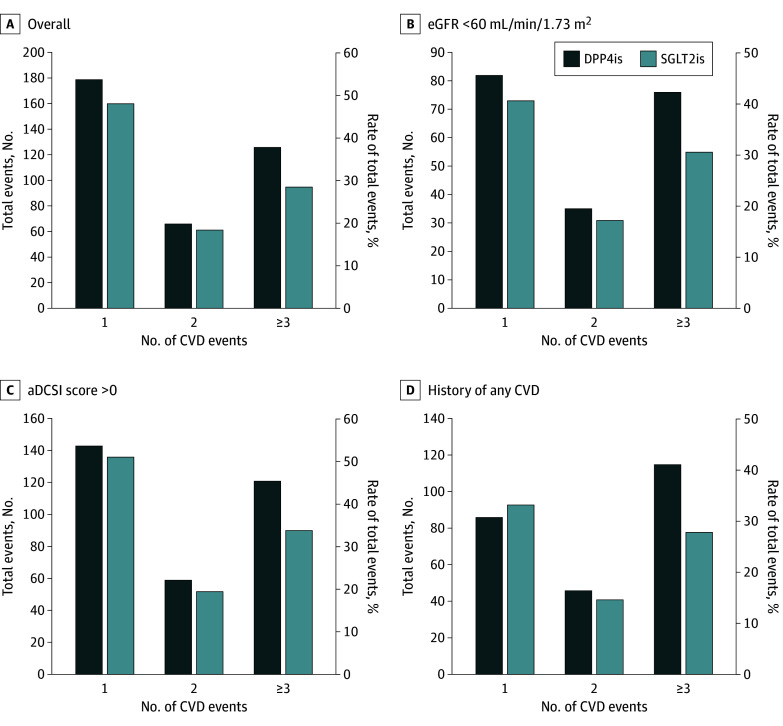
Distribution of Total Cardiovascular Disease (CVD) Events by Number of Events During Study Follow-Up for the Overall Cohort and 3 Subgroups Percentages were calculated as the number of CVD events in each category divided by the total number of CVD events in each treatment group during the follow-up period. aDCSI represents adapted Diabetes Complications Severity Index; DPP4is, dipeptidyl peptidase 4 inhibitors; eGFR, estimated glomerular filtration rate; HR, hazard ratio; and SGLT2is, sodium-glucose cotransporter 2 inhibitors.

**Figure 3.**
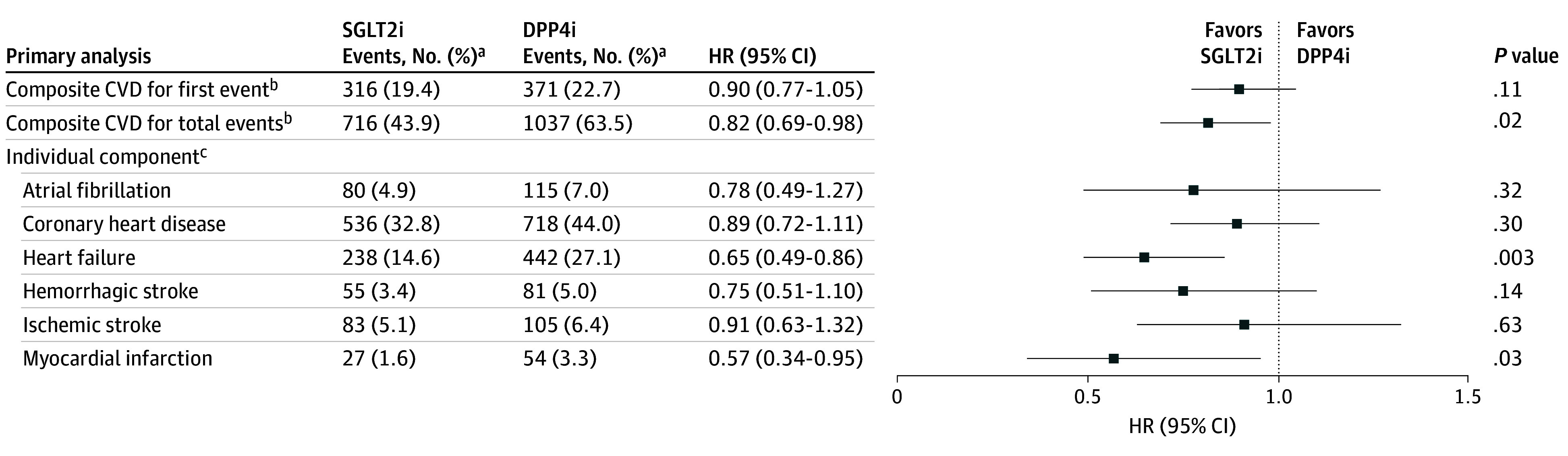
Risk for Time to First Cardiovascular Disease (CVD) Event (Using Cox Proportional Hazards Model Analysis) and Total CVD Events (Using Shared Frailty Model Analysis) Associated With SGLT2i vs DPP4i Use in Overall Patient Cohort (N = 1632) DPP4is represents dipeptidyl peptidase 4 inhibitors: SGLT2is, sodium-glucose cotransporter 2 inhibitors. ^a^Events are the first or repeat CVD events, and rates (%) were calculated as the total number of events divided by the total number of patients in each treatment group. ^b^Composite CVD included atrial fibrillation, coronary heart disease, heart failure, hemorrhagic stroke, ischemic stroke, myocardial infarction, and transient ischemic attack. ^c^Since no recurrent transient ischemic attacks occurred in the study follow-up period, the analysis for this event was not performed.

**Figure 4.**
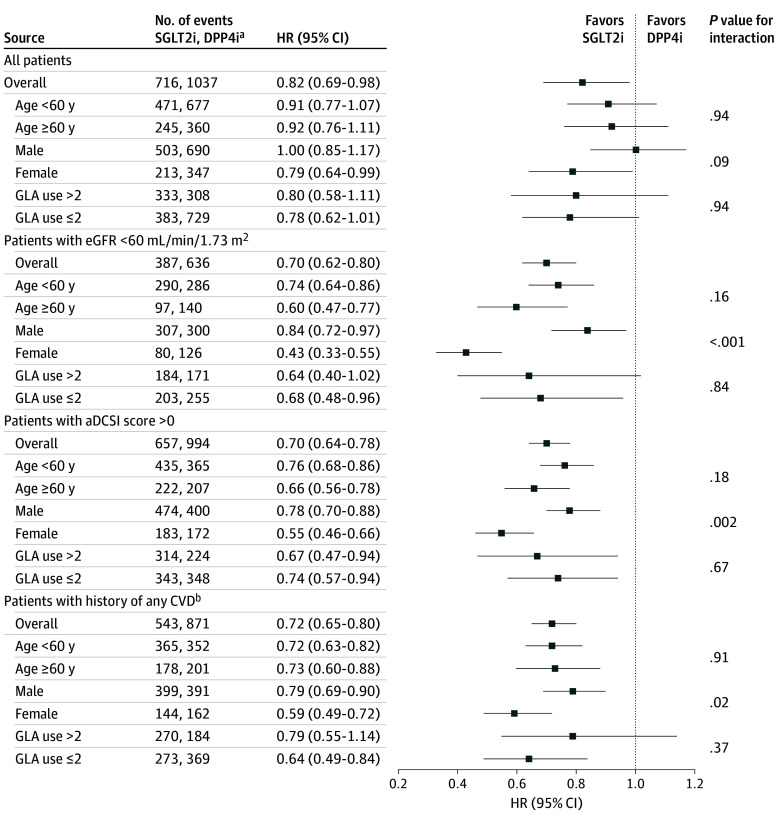
HRs for the Interaction of Treatment Exposure With Patient Baseline Characteristics in the Overall Cohort and Patient Subgroups GLA (glucose-lowering agent) includes acarbose, glucagon-like peptide-1 receptor agonists, insulin, meglitinides, metformin, sulfonylureas, and thiazolidinedione. aDCSI indicates adapted Diabetes Complications Severity Index; CVD, cardiovascular disease; DPP4is, dipeptidyl peptidase 4 inhibitors; eGFR, estimated glomerular filtration rate; HR, hazard ratio; and SGLT2is, sodium-glucose cotransporter 2 inhibitors. ^a^Events included the first and repeat CVD events. ^b^History of any CVD was determined from inpatient, outpatient, and emergency department medical records.

Differences in risks of the first composite CVD event with the use of SGLT2is vs DPP4is were not significant in the overall study cohort (hazard ratio [HR], 0.90 [95% CI, 0.77-1.05]) or across patient subgroups (HR, 0.87 [95% CI, 0.71-1.08] for eGFR lower than 60 mL/min/1.73m^2^; HR, 0.85 [0.73-1.00] for aDCSI score higher than 0; and HR, 0.84 [95% CI, 0.70-1.01] for CVD history) (eTable 5 in Supplement 1). Figure 2 shows the distribution of total CVD events that occurred during the study follow-up period in the overall cohort and in patient subgroups stratified by SGLT2i and DPP4i users. In general, the use of SGLT2i was associated with lower CVD recurrence compared with the use of DPP4i. The recurrence rates (ie, having 2 or more CVDs during follow-up) in the overall cohort were 49.4% for SGLT2i use and 51.8% DPP4i use compared with 54.1% for SGLT2i use and 57.5% for DPP4i use in the subgroup with eGFR lower than 60 mL/min/1.73m^2^; 51.1% for SGLT2i use and 55.7% for DPP4i use in the subgroup with aDCSI score higher than 0; and 56.1% for SGLT2i use and 65.2% for DPP4i use in the subgroup with CVD history. Figure 3 shows that compared with the use of DPP4is, SGLT2i use was associated with a significantly reduced risk of total composite CVD events (HR, 0.82 [95% CI, 0.69-0.98]), HF (HR, 0.65 [95% CI, 0.49-0.86]), and MI (HR, 0.57 [95% CI, 0.34-0.95]). The subgroup analysis results were consistent with the primary findings, indicating decreased risk of total composite CVD associated with the use of SGLT2is vs DPP4is (HR, 0.70 [95% CI, 0.62-0.80] for eGFR lower than 60 mL/min/1.73m^2^; HR, 0.70 [95% CI, 0.64-0.78] for aDCSI score higher than 0; and HR, 0.72 [95% CI, 0.65-0.80] for CVD history) (Figure 4).

Figure 4 also shows the interaction testing results. No significant heterogeneity was found in the association of SGLT2i vs DPP4i therapy with CVD risks by these 3 characteristics (age at the index date, sex, and number of oral GLAs prescribed in the year before the index date) in the overall study cohort. However, significant interactions were observed for sex across the patient subgroups, with a greater benefit in reducing the total composite CVD risk associated with the use of SGLT2is vs DPP4is for female patients (HR, 0.43 [95% CI, 0.33-0.55] in the subgroup with eGFR lower than 60 mL/min/1.73m^2^; HR, 0.55 [95% CI, 0.46- 0.66] in the subgroup with aDCSI score higher than 0; and HR, 0.59 [95% CI, 0.49-0.72] in the subgroup with CVD history) compared with male patients (HR, 0.84 [95% CI, 0.72-0.97] in the subgroup with eGFR lower than 60 mL/min/1.73m^2^; HR, 0.78 [95% CI, 0.70-0.88] in the subgroup with aDCSI score higher than 0; and HR, 0.79 [95% CI, 0.69-0.90] in the subgroup with CVD history).

## Discussion

This cohort study is the first, to our knowledge, to provide clinical practice evidence regarding the effectiveness of SGLT2i vs DPP4i therapy associated with reduced risk of total (ie, first and subsequent) CVD events in a longitudinal cohort of patients with T2D (assessed from 2015 through 2021). Several methodologic efforts (eg, a shared frailty model analysis) (eDiscussion in Supplement 1) undertaken in this study not only ensured the study robustness but also enhanced the applicability of the study findings to clinical settings. Overall, the use of SGLT2is was associated with a markedly reduced risk of total CVD events compared with the risk of the first CVD event. Such an association with treatment benefit on all cardiovascular conditions was even more prominent among 3 patient subgroups (ie, eGFR lower than 60 mL/min/1.73m^2^, aDCSI score higher than 0, and history of CVD) and with specific patient characteristics (ie, female sex and comorbid CKD) associated with high risk for CVD recurrence. Hence, the results of this empirical study could extend trial efficacy findings obtained from selective and homogenous patient participants to diverse patient populations with T2D, suggesting the benefit of SGLT2i therapy associated with not only preventing development of the first cardiovascular event but also with averting the recurrence of events.

Substantial evidence from cardiovascular outcome trials has supported an association of a protective effect of SGLT2i therapy with time to first CVD event.^6,7,8^ However, given the chronic nature of both T2D and CVD, cardiovascular events could occur more than once, which is amplified by an individual’s age, prior CVD, and diabetes control and related complications (eg, CKD).^19^ Several post hoc analyses of cardiovascular outcome trials have thus analyzed repeat events, as a supplementary measure to time to first events,^20^ to determine the total treatment benefit of SGLT2is associated with the overall burden of chronic cardiovascular conditions over T2D disease progression. A numerically greater risk reduction in total events associated with the use of SGLT2is was found, compared with that of time to first CVD events.^6,7,8^ By expanding and integrating clinical research into more diverse, clinical practice settings, the present study using data from real-world clinical practice showed that receipt of SGLT2i therapy compared with DPP4i therapy was associated with a significantly reduced risk of total composite CVD (HR, 0.82 [95% CI, 0.69-0.98]) according to the shared frailty model analysis, but was not associated with a significantly decreased time to first CVD event (HR, 0.90 [95% CI, 0.77-1.05]) based on traditional Cox proportional hazards model analysis. This finding may be explained by a potential gain in the statistical power and efficiency of recurrent analyses, in which subsequent events are accounted for, compared with traditional Cox model analyses, which consider only a single event per person.^5^ However, an observational study assessing empagliflozin vs sitagliptin therapy among an elderly population with diabetes revealed fairly comparable estimates for the associations of the treatments with the first and recurrent events across different components of CVD (eg, HF hospitalization, MI, or stroke), which may be due to the low recurrence of events in the relatively short study follow-up period (ie, approximately 6 months).^21^

The present study measured cardiovascular outcomes that comprised several clinically meaningful subtypes of CVD (ie, HF, MI, stroke, CHD, and AF) to better characterize the association of SGLT2i use with the overall disease burden. Of note, a treatment benefit associated with SGLT2i use was not apparent for all CVD subtypes. Specifically, the risks were lower for MI (HR, 0.57 [95% CI, 0.34-0.95]) and HF (HR, 0.65 [95% CI, 0.49-0.86]) compared with other subtypes (HR, 0.91 [95% CI, 0.63-1.32] for stroke; HR, 0.89 [95% CI, 0.72-1.11] for CHD; and HR, 0.78 [95% CI, 0.49-1.27] for AF). These findings imply that SGLT2i therapy may be associated with benefits not only for HF but also for atherosclerotic cardiovascular conditions (eg, MI), which is in line with prior study findings.^12^

Diabetes and its severity, CKD, and CVD history are known to be independent risk factors for recurrent cardiovascular conditions.^22^ In particular, patients with both T2D and CKD have a greater risk of recurrent CVD than patients with CVD history or diabetes alone.^23^ In accordance with previous findings,^22,23^ the recurrent CVD event rate observed in this study was higher in patients at high risk for recurrence compared with the first CVD event rate (ie, 27% increase in CVD event [recurrent event, 197 vs time to first event, 155] among patients with eGFR lower than 60 mL/min/1.73m^2^; 15% increase [recurrent event, 322 vs time to first event, 279] among patients with aDCSI score higher than 0; and 56% increase [recurrent event, 280 vs time to first event, 179] among patient with a history of CVD), suggesting an urgency for alleviating the excess CVD burden in these vulnerable patients with T2D. Moreover, the risk reduction associated with SGLT2i use for the total CVD burden among these patients was numerically greater than that for the general T2D study cohort (ie, risk reduction, 30% and HR, 0.70 [95% CI, 0.62-0.80] for patients with eGFR lower than 60 mL/min/1.73m^2^; risk reduction, 30% and HR, 0.70 [95% CI, 0.64-0.78] for aDCSI score higher than 0; and risk reduction, 28% and HR, 0.72 [95% CI, 0.65-0.80] for CVD history; vs risk reduction, 18% and HR, 0.82 [95% CI, 0.69-0.98] for the overall study cohort). Hence, these results support the important therapeutic role of SGLT2is for these patients, which is consistent with many treatment guidelines that endorse the prioritization of SGLT2is for these high-risk populations to reduce CVD risk.^9,10,11^ Unfortunately, SGLT2is remain underprescribed for these patients at high risk in routine practice,^24^ and this underprescribing deserves greater attention from individual patients, clinicians, and health care systems.

Furthermore, among these patients at high-risk for CVD, the association of SGLT2i therapy with the risk of the total CVD burden was more prominent for female patients than for male patients, suggesting a heterogeneous association of treatments with outcomes by sex. This finding was expected given the 40% higher risk of CVD for women with diabetes than for men with diabetes shown in a previous study.^25^ In addition, it is known that women with diabetes comorbid with CKD have a greater excess risk of recurrent cardiovascular conditions compared with men with this comorbidity.^23^ This may explain our finding that among patients with T2D with eGFR lower than 60 mL/min/1.73m^2^, the HR of SGLT2i treatment associated with reduced CVD events was lower for women than for men (HR, 0.43 [95% CI, 0.33-0.55] vs HR, 0.84 [95% CI, 0.72-0.97]; *P* < .001 for interaction). These potential heterogeneous associations of SGLT2is with outcomes by demographic (ie, sex) and clinical (ie, comorbid CKD) characteristics suggest a need for personalized treatments tailored by relevant patient characteristics to maximize treatment benefits.

### Limitations

First, similar to other observational studies, residual unmeasured confounding (eg, diabetes duration) may not have been excluded in the present study. However, several variables were assessed as proxies for diabetes severity (ie, HbA_1c_, diabetes-related complications, and previous exposure to GLAs) and adjusted using PSM. Satisfactory between-group comparability in these variables suggested similar diabetes severity in study patients. Consistent results for the reduced risk of time to first CVD event in the positive control outcome analysis of this study and previous studies suggest that the potential unmeasured confounding in this study was minimal.^6,7,8^ Since multiple states (ie, recurrent events) and their transitions are considered in the analyses, a multistate model approach can be applied in future research to corroborate our findings.^26^ Second, since this study retrieved data from the EMRs of a health care system, the continuity of health care services may not have been ensured. However, NCKUH is a leading medical center in Taiwan, and the continuity of health care among patients with T2D in this system was acceptable (ie, 64% and 74% of patients with T2D had continuous records during 6-month baseline and 24-month follow-up periods, respectively),^27^ implying that our study cohort was more likely to return to NCKUH; thus, the problem of health care continuity may be negligible. Third, similar to other studies assessing populations from clinical practice, short-term exposure to the drug of interest may not have been eliminated in our study populations, resulting in a concern of treatment misclassification. However, this concern may have been minimized owing to the inclusion of only stable users of the study drugs in our analysis. Fourth, due to the unavailability of death records in the EMRs of NCKUH, cardiovascular deaths could not be assessed as a study outcome. Fifth, subgroup analyses were not conducted according to the presence of diabetic retinopathy or neuropathy due to a limited number of study patients with these diabetes-related complications. Considering the importance of these complications for patients with T2D, future research is encouraged to stratify the analysis by the presence of these complications. Lastly, effectiveness was presented as HRs in our study, which does not imply that causality can be inferred from our findings.

## Conclusions

In this cohort study of patients with T2D, a significantly reduced risk of total CVD was associated with SGLT2i vs DPP4i therapy; that reduction was lower than that for time to first CVD event. This lower risk for the association between SGLT2i therapy and the total CVD burden was most prominent among patients at high risk for CVD recurrence. Hence, the prioritization of SGLT2i therapy for this vulnerable population should be encouraged, with long-term use for a potential benefit on the chronic cardiovascular burden.

## Notes

Supplement 1.
eMethods.
**eFigure.** Flow chart for patient selection **eTable 1.** Baseline patient characteristics before and after propensity score matching in overall study patients **eTable 2.** Baseline patient characteristics before and after propensity score matching stratified by gender **eTable 3.** International Classification of Disease, 9th and 10th editions, Clinical Modification (ICD-9-CM and ICD-10-CM) codes for cardiovascular events of interest **eTable 4.** Baseline patient characteristics before and after propensity score matching among patient subgroups **eTable 5.** Results of risk of composite cardiovascular diseases associated with SGLT2i versus DPP4i use using traditional Cox proportional hazard model analyses in overall study cohort and patient subgroups
eDiscussion.

Supplement 2.
Data Sharing Statement

## References

[1] Mosenzon O, Alguwaihes A, Leon JLA, ; CAPTURE Study Investigators. CAPTURE: a multinational, cross-sectional study of cardiovascular disease prevalence in adults with type 2 diabetes across 13 countries. Cardiovasc Diabetol. 2021;20(1):154. doi:10.1186/s12933-021-01344-0 34315481 PMC8317423

[2] Einarson TR, Acs A, Ludwig C, Panton UH. Prevalence of cardiovascular disease in type 2 diabetes: a systematic literature review of scientific evidence from across the world in 2007-2017. Cardiovasc Diabetol. 2018;17(1):83. doi:10.1186/s12933-018-0728-6 29884191 PMC5994068

[3] Chen HY, Kuo S, Su PF, Wu JS, Ou HT. Health care costs associated with macrovascular, microvascular, and metabolic complications of type 2 diabetes across time: estimates from a population-based cohort of more than 0.8 million individuals with up to 15 years of follow-up. Diabetes Care. 2020;43(8):1732-1740. doi:10.2337/dc20-0072 32444454 PMC7372047

[4] Wilson PW, D’Agostino R Sr, Bhatt DL, ; REACH Registry. An international model to predict recurrent cardiovascular disease. Am J Med. 2012;125(7):695-703.e1. doi:10.1016/j.amjmed.2012.01.014 22727237

[5] Claggett B, Pocock S, Wei LJ, Pfeffer MA, McMurray JJV, Solomon SD. Comparison of time-to-first event and recurrent-event methods in randomized clinical trials. Circulation. 2018;138(6):570-577. doi:10.1161/CIRCULATIONAHA.117.033065 29588314

[6] Zinman B, Wanner C, Lachin JM, ; EMPA-REG OUTCOME Investigators. Empagliflozin, cardiovascular outcomes, and mortality in type 2 diabetes. N Engl J Med. 2015;373(22):2117-2128. doi:10.1056/NEJMoa1504720 26378978

[7] McMurray JJV, Solomon SD, Inzucchi SE, ; DAPA-HF Trial Committees and Investigators. Dapagliflozin in patients with heart failure and reduced ejection fraction. N Engl J Med. 2019;381(21):1995-2008. doi:10.1056/NEJMoa1911303 31535829

[8] Neal B, Perkovic V, Mahaffey KW, ; CANVAS Program Collaborative Group. Canagliflozin and cardiovascular and renal events in type 2 diabetes. N Engl J Med. 2017;377(7):644-657. doi:10.1056/NEJMoa1611925 28605608

[9] American Diabetes Association. 9. Pharmacologic approaches to glycemic treatment: standards of medical care in diabetes—2020. Diabetes Care. 2020;43(suppl 1):S98-S110. doi:10.2337/dc20-S009 31862752

[10] Das SR, Everett BM, Birtcher KK, . 2018 ACC expert consensus decision pathway on novel therapies for cardiovascular risk reduction in patients with type 2 diabetes and atherosclerotic cardiovascular disease: a report of the American College of Cardiology Task Force on Expert Consensus Decision Pathways. J Am Coll Cardiol. 2018;72(24):3200-3223. doi:10.1016/j.jacc.2018.09.020 30497881 PMC7560953

[11] Garber AJ, Handelsman Y, Grunberger G, . Consensus statement by the American Association of clinical Endocrinologists and American College Of Endocrinology on the comprehensive type 2 diabetes management algorithm—2020 executive summary. Endocr Pract. 2020;26(1):107-139. doi:10.4158/CS-2019-0472 32022600

[12] McGuire DK, Zinman B, Inzucchi SE, . Effects of empagliflozin on first and recurrent clinical events in patients with type 2 diabetes and atherosclerotic cardiovascular disease: a secondary analysis of the EMPA-REG OUTCOME trial. Lancet Diabetes Endocrinol. 2020;8(12):949-959. doi:10.1016/S2213-8587(20)30344-2 33217335

[13] Jhund PS, Ponikowski P, Docherty KF, . Dapagliflozin and recurrent heart failure hospitalizations in heart failure with reduced ejection fraction: an analysis of DAPA-HF. Circulation. 2021;143(20):1962-1972. doi:10.1161/CIRCULATIONAHA.121.053659 33832352 PMC8126492

[14] Li JW, Arnott C, Heerspink HJL, . Effect of canagliflozin on total cardiovascular burden in patients with diabetes and chronic kidney disease: a post hoc analysis from the CREDENCE trial. J Am Heart Assoc. 2022;11(16):e025045. doi:10.1161/JAHA.121.025045 35929472 PMC9496296

[15] Zelniker TA, Bonaca MP, Furtado RHM, . Effect of dapagliflozin on atrial fibrillation in patients with type 2 diabetes mellitus: insights from the DECLARE-TIMI 58 trial. Circulation. 2020;141(15):1227-1234. doi:10.1161/CIRCULATIONAHA.119.044183 31983236

[16] National Cheng Kung University Hospital. 2022 Annual report of the National Cheng Kung University Hospital. Accessed March 19, 2024. https://nckuh.hosp.ncku.edu.tw/p/412-1000-29534.php#gsc.tab=0

[17] Desai JR, Hyde CL, Kabadi S, . Utilization of positive and negative controls to examine comorbid associations in observational database studies. Med Care. 2017;55(3):244-251. doi:10.1097/MLR.0000000000000640 27787351 PMC5318155

[18] Therneau TM, Grambsch PM, Pankratz VS. Penalized survival models and frailty. J Comput Graph Stat. 2003 Mar;12(1):156-175.

[19] Giorda CB, Avogaro A, Maggini M, ; Diabetes and Informatics Study Group. Recurrence of cardiovascular events in patients with type 2 diabetes: epidemiology and risk factors. Diabetes Care. 2008;31(11):2154-2159. doi:10.2337/dc08-1013 18782902 PMC2571066

[20] Gregson J, Stone GW, Bhatt DL, . Recurrent events in cardiovascular trials: JACC state-of-the-art review. J Am Coll Cardiol. 2023;82(14):1445-1463. doi:10.1016/j.jacc.2023.07.024 37758440

[21] Desai RJ, Glynn RJ, Everett BM, . Comparative effectiveness of empagliflozin in reducing the burden of recurrent cardiovascular hospitalizations among older adults with diabetes in routine clinical care. Am Heart J. 2022;254:203-215. doi:10.1016/j.ahj.2022.09.008 36150454

[22] Mondesir FL, Brown TM, Muntner P, . Diabetes, diabetes severity, and coronary heart disease risk equivalence: reasons for geographic and racial differences in stroke (REGARDS). Am Heart J. 2016;181:43-51. doi:10.1016/j.ahj.2016.08.002 27823692 PMC5117821

[23] Hubbard D, Colantonio LD, Rosenson RS, . Risk for recurrent cardiovascular disease events among patients with diabetes and chronic kidney disease. Cardiovasc Diabetol. 2021;20(1):58. doi:10.1186/s12933-021-01247-0 33648518 PMC7923492

[24] Hussain A, Ramsey D, Lee M, . Utilization rates of SGLT2 inhibitors among patients with type 2 diabetes, heart failure, and atherosclerotic cardiovascular disease: insights from the Department of Veterans Affairs. JACC Heart Fail. 2023;11(8 Pt 1):933-942. doi:10.1016/j.jchf.2023.03.024 37204363

[25] Peters SA, Huxley RR, Woodward M. Diabetes as risk factor for incident coronary heart disease in women compared with men: a systematic review and meta-analysis of 64 cohorts including 858,507 individuals and 28,203 coronary events. Diabetologia. 2014;57(8):1542-1551. doi:10.1007/s00125-014-3260-6 24859435

[26] Amorim LD, Cai J. Modelling recurrent events: a tutorial for analysis in epidemiology. Int J Epidemiol. 2015;44(1):324-333. doi:10.1093/ije/dyu222 25501468 PMC4339761

[27] Hsu CN, Huang K, Lin FJ, . Continuity and completeness of electronic health record data for patients treated with oral hypoglycemic agents: findings from healthcare delivery systems in Taiwan. Front Pharmacol. 2022;13:845949. doi:10.3389/fphar.2022.845949 35444533 PMC9015706

