# Supplementary material for: Cardiovascular Risks With SGLT2 Inhibitors in Clinical Practice Among Patients With Type 2 Diabetes
Source: JAMA Netw Open. 2024 Oct 30;7(10):e2441765. doi: 10.1001/jamanetworkopen.2024.41765 (PMC11525605; doi:10.1001/jamanetworkopen.2024.41765)
Supplement: Supplement 1. — eMethods. eFigure. Flow chart for patient selection eTable 1. Baseline patient characteristics before and after propensity score matching in overall study patients eTable 2. Baseline patient characteristics before and after propensity score matching stratified by gender eTable 3. International Classification of Disease, 9th and 10th editions, Clinical Modification (ICD-9-CM and ICD-10-CM) codes for cardiovascular events of interest eTable 4. Baseline patient characteristics before and after propensity score matching among patient subgroups eTable 5. Results of risk of composite cardiovascular diseases associated with SGLT2i versus DPP4i use using traditional Cox proportional hazard model analyses in overall study cohort and patient subgroups eDiscussion. [file jamanetwopen-e2441765-s001.pdf]

Supplementary Online Content

Su HY, Yang CY, Lee YH, Su PF, Liu YC, Ou HT. Cardiovascular risks with SGLT2 inhibitors in clinical practice among patients with type 2 diabetes. *JAMA Netw Open*. 2024;7(10):e2441765. doi:10.1001/jamanetworkopen.2024.41765

eMethods.
eFigure 1. Flow chart for patient selection
eTable 1. Baseline patient characteristics before and after propensity score matching in overall study patients
eTable 2. Baseline patient characteristics before and after propensity score matching stratified by sex
eTable 3. International Classification of Disease, 9th and 10th editions, Clinical Modification (ICD-9-CM and ICD-10-CM) codes for cardiovascular events of interest
eTable 4. Baseline patient characteristics before and after propensity score matching among patient subgroups
eTable 5. Results of risk of composite cardiovascular diseases associated with SGLT2i versus DPP4i use using traditional Cox proportional hazard model analyses in overall study cohort and patient subgroups
eDiscussion.

This supplementary material has been provided by the authors to give readers additional information about their work.

© 2024 Su HY et al. *JAMA Network Open*.

eMethods.

Study cohort identification

1. New users of dipeptidyl peptidase 4 inhibitors (DPP4is) or sodium-glucose cotransporter 2 inhibitors (SGLT2is) were defined as patients without any exposure to these drugs in the 3 months before the index date.

2. Stable users were defined as having at least three sequential refills of DPP4is or SGLT2is with a gap between two consecutive prescriptions of less than 30 days.

3. Demographics (i.e., age and sex), comorbidities (i.e., myocardial infarction, ischemic stroke, hemorrhagic stroke, heart failure, transient ischemic stroke, coronary heart disease, atrial fibrillation, and Charlson comorbidity index), laboratory data (i.e., total cholesterol, high-density lipoprotein, low-density lipoprotein, triglycerides, glycated hemoglobin), diabetes-related complications (i.e., adapted Diabetes Complications Severity Index), other glucose-lowering agents (GLAs) (i.e., acarbose, glucagon-like peptide-1 receptor agonist, insulin, meglitinides, metformin, sulfonylureas, thiazolidinedione), and cardiovascular-related medications (i.e., alpha blocker, antiarrhythmics, anticoagulants, antiplatelet agents, beta blocker, calcium channel blocker, digoxin, diuretics, lipid modifying agents, renin-angiotensin-aldosterone system, and vasodilators) that were associated with treatment exposure or study outcomes were measured in the year before the index date to estimate the propensity score of individuals using a logistic regression model analysis.

4. The standardized mean difference (SMD) was used to assess between-treatment-group comparability before and after the matching, with an SMD value of above 0.1 indicating a statistical imbalance in patient characteristics.

© 2024 Su HY et al. *JAMA Network Open*.

eFigure 1. Flow chart for patient selection

Stable drug users* between January 2016 – June 2019

- DPP4is: 12,111 patients
- SGLT2is: 2,642 patients

Excluding:

- No laboratory exam in the year prior to the index date† (n=5,069)
- Patients who had both newly stable use of DPP4is and SGLT2is at the same day (i.e., at the same index date) or exposure to either DPP4is or SGLT2is in 3 months prior to the index date (n=1,042)
- Exposure to comparison treatments‡ in 180 days after the index date (n=258)

Before PS matching

- DPP4is: 6,752 patients
- SGLT2is: 1,632 patients

After 1:1 PS matching

- DPP4is: 1,632 patients
- SGLT2is: 1,632 patients

Abbreviations: SGLT2is, sodium-glucose co-transporter 2 inhibitors; DPP4is, dipeptidyl peptidase 4 inhibitors.

Notes:

*Stable use was defined as patients with at least three prescriptions of study drug (i.e., SGLT2is or DPP4is) with any gaps between two consecutive drug refills of less than 30 days.

†Index date refers to the date of initiation of SGLT2i or DPP4i treatment.

‡Comparison treatments refers to either SGLT2is for DPP4i users or DPP4is for SGLT2i users.

© 2024 Su HY et al. *JAMA Network Open*.

eTable 1. Baseline patient characteristics before and after propensity score matching in overall study patients

	Before matching		After matching	
**Baseline characteristics**	**SGLT2is**	**DPP4is**	**SGLT2is**	**DPP4is**
Number of cases	1,632	6,752	1,632	1,632
Age at index date† (year, mean±SD)	57.8 ± 12.0	65.2 ± 12.1*	57.8 ± 12.0	58.2 ± 12.9
Follow-up (until the first study event occurred) (year, mean±SD)	2.9 ± 1.9	3.2 ± 2.4	2.9 ± 1.9	3.3 ± 2.3
Follow-up (until the end of 2021) (year, mean±SD)	4.1 ± 0.8	5.1 ± 1.0	4.1 ± 0.8	4.8 ± 1.0
Female (%)	41.2	45.4	41.2	40.1
**CVD history‡ in the year before index date (%)**				
MI	3.1	2.7	3.1	3.6
Stroke				
Ischemic stroke	4.5	8.4*	4.5	4.2
Hemorrhagic stroke	1.4	2.9*	1.4	1.7
Heart failure	7.2	8.2	7.2	7.5
TIA	0.4	0.7	0.4	0.2
CHD (not including AMI)	21.2	21.9	21.2	21.0
AF	2.3	3.9	2.3	2.5
**Comorbidity history (measured by CCI) in the year before/at index date (mean±SD)**	2.9 ± 1.8	2.9 ± 1.9	2.9 ± 1.8	2.8 ± 1.9
**Laboratory exam in the year before/at index date (mean±SD)**				
TC	163.1 ± 34.4	164.2 ± 38.2	163.1 ± 34.4	163.2 ± 37.2
HDL	46.0 ± 13.1	47.1 ± 13.7	46.0 ± 13.1	46.2 ± 14.3
LDL	100.5 ± 28.2	101.6 ± 31.4	100.5 ± 28.2	100.8 ± 30.1

© 2024 Su HY et al. *JAMA Network Open*.

Triglycerides	102.5 ± 143.4	83.8 ± 116.1*	102.5 ± 143.4	96.5 ± 127.5
$HbA1_c$	8.6 ± 1.4	7.9 ± 1.4*	8.6 ± 1.4	8.5 ± 1.8
**Diabetes-related complications (measured by aDCSI) in the year before index date (mean±SD)**	1.5 ± 1.4	0.8 ± 1.5*	1.5 ± 1.4	1.6 ± 1.8
**Glucose-lowering agents in the year before and at index date (measured by MPR, presented as days) (mean±SD)**				
Acarbose	28.1 ± 74.5	14.5 ± 54.9*	28.1 ± 74.5	24.9 ± 75.2
GLP-1RA	8.1 ± 43.6	0.4 ± 7.9*	8.1 ± 43.6	4.4 ± 35.8
Insulin	59.9 ± 106	27.4 ± 74.4*	59.9 ± 106	51.3 ± 102.8
Meglitinides	8.0 ± 42.7	12.6 ± 50.5	8.0 ± 42.7	8.2 ± 41.3
Metformin	163.8 ± 111.7	123.9 ± 113.7*	163.8 ± 111.7	158.1 ± 120.3
Sulfonylureas	106.2 ± 116	87.0 ± 110.9*	106.2 ± 116	103.1 ± 122.8
TZD	20.5 ± 61.2	7.1 ± 37.0*	20.5 ± 61.2	15.1 ± 58.1
**Medication history in the year before and at index date (CVD related) (%)**				
Alpha blockers	2.1	4.8*	2.1	2.0
Antiarrhythmics	6.9	9.6	6.9	7.0
Anticoagulants	3.5	3.9	3.5	3.4
Antiplatelet agents	31.2	38.8*	31.2	32.4
Beta blockers	29.8	32.0	29.8	30.1
CCB	38.7	48.1*	38.7	39.0
Digoxin	1.5	1.5	1.5	1.7
Diuretics	14.7	23.9*	14.7	15.3
Lipid modifying agents	77.5	71.5*	77.5	76.8
RAAS agents	50.4	48.8	50.4	48.6

© 2024 Su HY et al. *JAMA Network Open*.

Vasodilators	17.3	19.4	17.3	17.5

Abbreviations: SGLT2is, sodium-glucose co-transporter 2 inhibitors; DPP4is, dipeptidyl peptidase 4 inhibitors; SD, standard deviation, CVD, cardiovascular disease; MI, myocardial infarction; TIA, transient ischemic attack; CHD, coronary heart disease; AMI, acute myocardial infarction; AF, atrial fibrillation; CCI, Charlson comorbidity index; TC, total cholesterol, HDL, high density lipoprotein; LDL, low density lipoprotein; HbA1c, glycated haemoglobin; aDCSI, adapted diabetes complications severity index; MPR, medication possession ratio; GLP-1RA, glucagon -like peptide-1; TZD, Thiazolidinedione, CCB, calcium channel blocker, RAAS, renin-angiotensin-aldosterone system.

Notes:

*An absolute standardized mean difference (SMD) > 0.1 indicates a significant between-group difference in baseline characteristics.

†Index date refers to the date of newly stable SGLT2i or DPP4i use in the study period.

‡CVD history was determined by patients who experienced any CVD events according to inpatient, outpatient and emergency records.

© 2024 Su HY et al. *JAMA Network Open*.

eTable 2. Baseline patient characteristics before and after propensity score matching stratified by sex

	Before matching		After matching	
**Baseline characteristics**	**Male**	**Female**	**Male**	**Female**
Number of cases	4,645	3,739	1,936	1,328
Age at index date† (year, mean±SD)	62.4 ± 12.2	65.3 ± 12.5*	57.4 ± 12.2	58.9 ± 12.8*
Follow-up (until the first study event occurred) (year, mean±SD)	2.9 ± 2.4	3.5 ± 2.3*	3.8 ± 1.6	4.0 ± 1.5*
Follow-up (until the end of 2021) (year, mean±SD)	4.9 ± 1.0	5.0 ± 1.0	4.4 ± 1.0	4.5 ± 1.0
SGLT2is (%)	20.6	18.0	49.5	50.7
**CVD history‡ in the year before index date (%)**				
MI	3.6	1.7*	4.5	1.6*
Stroke				
Ischemic stroke	8.8	6.2	4.9	3.5
Hemorrhagic stroke	2.9	2.2	1.7	1.4
Heart failure	8.7	7.1	8.4	5.8*
TIA	.6	.7	.2	.6
CHD (not including AMI)	26.2	16.3*	25.9	14.0*
AF	4.0	3.0	2.9	1.7
**Comorbidity history (measured by CCI) in the year before/at index date (mean±SD)**	2.9 ± 1.9	2.9 ± 1.8	2.9 ± 1.8	2.9 ± 1.8
**Laboratory exam in the year before/at index date (mean±SD)**				
TC	160.4 ± 38.2	168.4 ± 36.1*	160.8 ± 36.7	166.5 ± 34.3*
HDL	43.8 ± 12.6	50.7 ± 13.9*	43.3 ± 12.6	50.3 ± 14.2*
LDL	100.0 ± 30.5	103.0 ± 31.0	99.7 ± 28.5	102.0 ± 30.0
Triglycerides	93.0 ± 129.1	80.4 ± 112.4*	107.0 ± 144.5	88.5 ± 120.9*

© 2024 Su HY et al. *JAMA Network Open*.

HbA1c	8.1 ± 1.5	8.1 ± 1.4	8.5 ± 1.7	8.6 ± 1.5
**Diabetes-related complications (measured by aDCSI) in the year before index date (mean±SD)**	1.0 ± 1.5	.9 ± 1.4	1.6 ± 1.7	1.4 ± 1.6
**Glucose-lowering agents in the year before and at index date (measured by MPR, presented as days) (mean±SD)**				
Acarbose	17.3 ± 59.9	17.0 ± 58.8	26.2 ± 74.9	27.0 ± 74.9
GLP-1RA	1.7 ± 19.6	2.1 ± 22.1	4.0 ± 30.1	5.9 ± 36.7
Insulin	33.6 ± 82.6	34.0 ± 82.5	52.4 ± 102.7	57.5 ± 105.9
Meglitinides	11.5 ± 49.7	11.9 ± 48.5	7.4 ± 40.8	9.2 ± 43.7
Metformin	130.2 ± 114.4	133.6 ± 114.3	157.7 ± 114.9	165.7 ± 117.6
Sulfonylureas	88.8 ± 111.4	93.3 ± 113.1	102.1 ± 117.1	108.4 ± 122.7
TZD	9.1 ± 42.0	10.4 ± 44.4	16.1 ± 57.5	20.3 ± 62.8
**Medication history in the year before and at index date (CVD related) (%)**				
Alpha blockers	4.1	4.5	1.8	2.4
Antiarrhythmics	8.4	9.9	5.8	8.6*
Anticoagulants	3.9	3.7	3.5	3.4
Antiplatelet agents	36.8	38.1	31.1	32.8
Beta blockers	31.3	32.0	29.8	30.3
CCB	45.4	47.3	37.9	40.2
Digoxin	1.5	1.5	1.5	1.7
Diuretics	21.8	22.5	14.5	15.7
Lipid modifying agents	72.2	73.3	76.2	78.5
RAAS agents	48.9	49.3	49.6	49.3
Vasodilators	18.9	19.1	17.0	17.9

Abbreviations: SGLT2is, sodium-glucose co-transporter 2 inhibitors; DPP4is, dipeptidyl peptidase 4 inhibitors; SD, standard deviation, CVD, cardiovascular disease; MI, myocardial infarction; TIA, transient ischemic

© 2024 Su HY et al. *JAMA Network Open*.

attack; CHD, coronary heart disease; AMI, acute myocardial infarction; AF, atrial fibrillation; CCI, Charlson comorbidity index; TC, total cholesterol, HDL, high density lipoprotein; LDL, low density lipoprotein; HbA1c, glycated haemoglobin; aDCSI, adapted diabetes complications severity index; MPR, medication possession ratio; GLP-1RA, glucagon -like peptide-1; TZD, Thiazolidinedione, CCB, calcium channel blocker, RAAS, renin-angiotensin-aldosterone system.

Notes:

*An absolute standardized mean difference (SMD) > 0.1 indicates a significant between-group difference in baseline characteristics.

†Index date refers to the date of newly stable SGLT2i or DPP4i use in the study period.

‡CVD history was determined by patients who experienced any CVD events according to inpatient, outpatient and emergency records.

© 2024 Su HY et al. *JAMA Network Open*.

eTable 3. International Classification of Disease, 9th and 10th editions, Clinical Modification (ICD-9-CM and ICD-10-CM) codes for cardiovascular events of interest

	ICD-9-CM disease code	ICD-10-CM disease code	Data source
Atrial fibrillation	427.31	I45	ER or inpatient
Coronary heart disease	411, 412, 413, 414	I20, I22, I23, I24, I25	ER or inpatient
Heart failure	428	I50	ER or inpatient
Hemorrhagic stroke	430, 431, 432	I60, I61, I62	ER or inpatient
Ischemic stroke	433, 434	I63	ER or inpatient
Myocardial infarction	410	I121	ER or inpatient
Transient ischemic attack	435,	G45, G46.0, G46.1, G46.2, I67.84	ER or inpatient

Abbreviation: ER, emergency room.

Reference: *Diabetes Obes Metab.* 2022 Aug;24(8):1623-1637.

© 2024 Su HY et al. *JAMA Network Open*.

eTable 4. Baseline patient characteristics before and after propensity score matching among patient subgroups

(a) eGFR <60mL/min/1.73m^2 at one year before index date

	Before matching		After matching	
**Baseline characteristics**	**SGLT2is**	**DPP4is**	**SGLT2is**	**DPP4is**
Number of cases	585	3,646	585	585
Age at index date† (year, mean±SD)	63.3 ± 10.5	68.8 ± 11*	63.3 ± 10.5	63.5 ± 11.3
Follow-up (until the first study event occurred) (year, mean±SD)	2.4 ± 2.0	2.9 ± 2.5	2.4 ± 2.0	2.8 ± 2.4
Follow-up (until the end of 2021) (year, mean±SD)	4.1 ± 0.8	5.2 ± 0.9	4.1 ± 0.8	4.9 ± 1
Female (%)	31.6	41.4*	31.60	30.10
**CVD history‡ in the year before index date (%)**				
MI	4.6	3.2	4.6	5.10
Stroke				
Ischemic stroke	7.0	9.2	7.0	7.2
Hemorrhagic stroke	1.4	2.6	1.4	1.5
Heart failure	10.1	11.7	10.1	10.4
TIA	0.5	0.6	0.5	0.5
CHD (not including AMI)	27.9	28.0	27.9	28.7
AF	4.3	5.2	4.3	5
**Comorbidity history (measured by CCI) in the year before/at index date (mean±SD)**	3.4 ± 1.9	3.4 ± 1.9	3.4 ± 1.9	3.4 ± 1.9
**Laboratory exam in the year before/at index date (mean±SD)**				
TC	159.5 ± 33.1	163.2 ± 39.8*	159.5 ± 33.1	162.6 ± 41.5
HDL	44.2 ± 12.1	46.1 ± 13.8*	44.2 ± 12.1	44.5 ± 13.4

© 2024 Su HY et al. *JAMA Network Open*.

	Before matching		After matching	
LDL	97.7 ± 26.7	100.2 ± 32.3	97.7 ± 26.7	100.9 ± 34.4
Triglycerides	112.8 ± 157.1	88.5 ± 118.5*	112.8 ± 157.1	114.4 ± 139.7
$HbA1_c$	8.6 ± 1.5	7.8 ± 1.4*	8.6 ± 1.5	8.5 ± 1.8
**Diabetes-related complications (measured by aDCSI) in the year before index date (mean±SD)**	2.0 ± 1.5	1.1 ± 1.7*	2 ± 1.5	2.2 ± 2.1
**Glucose-lowering agents in the year before and at index date (measured by MPR, presented as days) (mean±SD)**				
Acarbose	26.9 ± 72.6	14.6 ± 55.5*	26.9 ± 72.6	26.7 ± 79.4
GLP-1RA	8.2 ± 43.8	0.4 ± 9.2*	8.2 ± 43.8	4 ± 36.9
Insulin	63.9 ± 107.6	29.2 ± 77.4*	63.9 ± 107.6	57.4 ± 110.8
Meglitinides	7.1 ± 38.5	12.8 ± 51.3*	7.1 ± 38.5	6.3 ± 35.9
Metformin	157.2 ± 110.7	125.2 ± 114.8*	157.2 ± 110.7	151 ± 124.9
Sulfonylureas	105.8 ± 113.6	86.2 ± 110.5*	105.8 ± 113.6	107.8 ± 127.4
TZD	19.4 ± 60.3	6.4 ± 35.1*	19.4 ± 60.3	13.7 ± 59.4
**Medication history in the year before and at index date (CVD related) (%)**				
Alpha blockers	2.4	5.0*	2.4	2.7
Antiarrhythmics	7.0	9.1	7.0	6.7
Anticoagulants	4.3	3.6	4.3	3.8
Antiplatelet agents	33	39.3*	33.0	34.7
Beta blockers	33.8	31.5	33.8	33.8
CCB	38.8	48.9*	38.8	39.0
Digoxin	1.7	1.2	1.7	1.5
Diuretics	15.9	23.8*	15.9	15.2

© 2024 Su HY et al. *JAMA Network Open*.

	Before matching		After matching	
Lipid modifying agents	77.9	71.8*	77.9	77.6
RAAS agents	52.6	49.1	52.6	52.0
Vasodilators	20.0	19.6	20.0	20.5

Abbreviations: SGLT2is, sodium-glucose co-transporter 2 inhibitors; DPP4is, dipeptidyl peptidase 4 inhibitors; SD, standard deviation, CVD, cardiovascular disease; MI, myocardial infarction; TIA, transient ischemic attack; CHD, coronary heart disease; AMI, acute myocardial infarction; AF, atrial fibrillation; CCI, Charlson comorbidity index; TC, total cholesterol, HDL, high density lipoprotein; LDL, low density lipoprotein; HbA1c, glycated haemoglobin; aDCSI, adapted diabetes complications severity index; MPR, medication possession ratio; GLP-1RA, glucagon -like peptide-1; TZD, Thiazolidinedione, CCB, calcium channel blocker, RAAS, renin-angiotensin-aldosterone system.

Notes:

*An absolute standardized mean difference (SMD) > 0.1 indicates a significant between-group difference in baseline characteristics.

†Index date refers to the date of newly stable SGLT2i or DPP4i use in the study period.

‡CVD history was determined by patients who experienced any CVD events according to inpatient, outpatient and emergency records.

© 2024 Su HY et al. *JAMA Network Open*.

(b) aDCSI score >0 at one year before index date

	Before matching		After matching	
**Baseline characteristics**	**SGLT2is**	**DPP4is**	**SGLT2is**	**DPP4is**
Number of cases	1,062	2,165	1062	1062
Age at index date† (year, mean±SD)	59.4 ± 11.8	67 ± 12.2*	59.4 ± 11.8	60.5 ± 11.7
Follow-up (until the first study event occurred) (year, mean±SD)	2.4 ± 2.0	2.1 ± 2.1	2.4 ± 2.0	2.3 ± 2.1
Follow-up (until the end of 2021) (year, mean±SD)	4.1 ± 0.8	4.4 ± 0.9	4.1 ± 0.8	4.4 ± 0.9
Female (%)	39.3	43.6	39.3	40.1
**CVD history‡ in the year before index date (%)**				
MI	4.7	6.1	4.7	4.9
Stroke				
Ischemic stroke	6.9	15.2*	6.9	8.3
Hemorrhagic stroke	2.2	7.2*	2.2	2.3
Heart failure	10.9	16.1*	10.9	11.6
TIA	0.7	1.2	0.7	0.6
CHD (not including AMI)	32.5	33.1	32.5	34.1
AF	3.5	7.0*	3.5	4.2
**Comorbidity history (measured by CCI) in the year before/at index date (mean±SD)**	3.6 ± 1.7	4.0 ± 2.0*	3.6 ± 1.7	3.6 ± 1.8
**Laboratory exam in the year before/at index date (mean±SD)**				
TC	162.3 ± 34.9	167.5 ± 44.8*	162.3 ± 34.9	162.6 ± 38.3
HDL	45.3 ± 13	46 ± 13.6	45.3 ± 13.0	46.1 ± 13.9
LDL	99.1 ± 27.9	104.0 ± 36.0*	99.1 ± 27.9	99.5 ± 31.2

© 2024 Su HY et al. *JAMA Network Open*.

	Before matching		After matching	
Triglycerides	112.4 ± 155.2	89.2 ± 127.8*	112.4 ± 155.2	97.7 ± 130.9
$HbA1_c$	8.7 ± 1.5	8.0 ± 1.6*	8.7 ± 1.5	8.6 ± 1.7
**Diabetes-related complications (measured by aDCSI) in the year before index date (mean±SD)**	2.3 ± 1.1	2.6 ± 1.4*	2.3 ± 1.1	2.3 ± 1.2
**Glucose-lowering agents in the year before and at index date (measured by MPR, presented as days) (mean±SD)**				
Acarbose	28.4 ± 75.8	14.3 ± 54.8*	28.4 ± 75.8	21.9 ± 64.8
GLP-1RA	8.2 ± 43.5	0.3 ± 5.4*	8.2 ± 43.5	5.6 ± 7.6
Insulin	60.1 ± 105.8	25.8 ± 71.3*	60.1 ± 105.8	50 ± 86.6
Meglitinides	8.7 ± 44.4	12.8 ± 51.6	8.7 ± 44.4	9.5 ± 44.6
Metformin	164 ± 111.5	120 ± 113.7*	164 ± 111.5	152.3 ± 118.8
Sulfonylureas	105.5 ± 115.2	82.7 ± 110.4*	105.5 ± 115.2	96.9 ± 120.5
TZD	22.1 ± 64.0	6.8 ± 37.1*	22.1 ± 64.0	16.8 ± 48.5
**Medication history in the year before and at index date (CVD related) (%)**				
Alpha blockers	2.3	4.8*	2.3	2.9
Antiarrhythmics	7.0	9.7*	7.0	8.2
Anticoagulants	3.8	3.0	3.8	3.7
Antiplatelet agents	32.7	39.4*	32.7	34.0
Beta blockers	30.1	32.9	30.1	29.9
CCB	39.9	47.3*	39.9	40.6
Digoxin	1.8	1.2	1.8	1.9
Diuretics	15.4	24.3*	15.4	17.3
Lipid modifying agents	77.9	69.2*	77.9	76.6

© 2024 Su HY et al. *JAMA Network Open*.

	Before matching		After matching	
RAAS agents	51.0	47.3	51.0	49.2
Vasodilators	17.8	20.3	17.8	17.7

Abbreviations: SGLT2is, sodium-glucose co-transporter 2 inhibitors; DPP4is, dipeptidyl peptidase 4 inhibitors; SD, standard deviation, CVD, cardiovascular disease; MI, myocardial infarction; TIA, transient ischemic attack; CHD, coronary heart disease; AMI, acute myocardial infarction; AF, atrial fibrillation; CCI, Charlson comorbidity index; TC, total cholesterol, HDL, high density lipoprotein; LDL, low density lipoprotein; HbA1c, glycated haemoglobin; aDCSI, adapted diabetes complications severity index; MPR, medication possession ratio; GLP-1RA, glucagon -like peptide-1; TZD, Thiazolidinedione, CCB, calcium channel blocker, RAAS, renin-angiotensin-aldosterone system.

Notes:

*An absolute standardized mean difference (SMD) > 0.1 indicates a significant between-group difference in baseline characteristics.

†Index date refers to the date of newly stable SGLT2i or DPP4i use in the study period.

‡CVD history was determined by patients who experienced any CVD events according to inpatient, outpatient and emergency records.

© 2024 Su HY et al. *JAMA Network Open*.

(c) CVD history at one year before index date

	Before matching		After matching	
**Baseline characteristics**	**SGLT2is**	**DPP4is**	**SGLT2is**	**DPP4is**
Number of cases	458	2292	458	458
Age at index date† (year, mean±SD)	62 ± 10.9	68.9 ± 10.9*	62 ± 10.9	62.4 ± 11.3
Follow-up (until the first study event occurred) (year, mean±SD)	0.5 ± 1.3	0.5 ± 1.4	0.5 ± 1.3	0.6 ± 1.4
Follow-up (until the end of 2021) (year, mean±SD)	4 ± 0.8	5.2 ± 0.9	4 ± 0.8	4.8 ± 0.9
Female (%)	28.2	37.7*	28.2	31.0
**CVD history‡ in the year before index date (%)**				
MI	10.9	7.9*	10.9	12.9
Stroke				
Ischemic stroke	16.2	24.6*	16.2	17.9
Hemorrhagic stroke	5.0	8.5*	5.0	5.5
Heart failure	25.5	24.2	25.5	27.1
TIA	1.5	2.1	1.5	1.1
CHD (not including AMI)	75.5	64.6*	75.5	74.5
AF	8.1	11.4*	8.1	9.0
**Comorbidity history (measured by CCI) in the year before/at index date (mean±SD)**	3.4 ± 2.1	3.6 ± 2.1	3.4 ± 2.1	3.4 ± 2
**Laboratory exam in the year before/at index date (mean±SD)**				
TC	160.1 ± 34.4	159.7 ± 38.1	160.1 ± 34.4	160.9 ± 42.3
HDL	43.4 ± 11.2	44.7 ± 13.1*	43.4 ± 11.2	44.2 ± 13.4
LDL	98.3 ± 28.6	99.3 ± 32.2	98.3 ± 28.6	99.4 ± 32.8

© 2024 Su HY et al. *JAMA Network Open*.

	Before matching		After matching	
Triglycerides	108.6 ± 156.1	80.9 ± 107.1*	108.6 ± 156.1	103.1 ± 133.9
HbA1c	8.4 ± 1.4	7.9 ± 1.4*	8.4 ± 1.4	8.4 ± 1.8
**Diabetes-related complications (measured by aDCSI) in the year before index date** (**mean±SD**)	2.7 ± 1.5	1.6 ± 1.9*	2.7 ± 1.5	2.9 ± 2.0
**Glucose-lowering agents in the year before and at index date (measured by MPR, presented as days) (mean±SD)**				
Acarbose	25.3 ± 71.5	12.8 ± 51.7*	25.3 ± 71.5	20.7 ± 71.1
GLP-1RA	8.9 ± 46.5	0.2 ± 4.6*	8.9 ± 46.5	5.5 ± 10.2
Insulin	58.3 ± 104.7	27.5 ± 74.9*	58.3 ± 104.7	48.4 ± 99.1
Meglitinides	9.8 ± 47.8	13.4 ± 53.1	9.8 ± 47.8	11.6 ± 50.9
Metformin	155.6 ± 109.8	123.7 ± 113.9*	155.6 ± 109.8	147.5 ± 119.7
Sulfonylureas	93.7 ± 109.9	85 ± 108.8	93.7 ± 109.9	96.4 ± 117.7
TZD	19.3 ± 55.3	6.1 ± 34.4*	19.3 ± 55.3	13.5 ± 57.2
**Medication history in the year before and at index date (CVD related) (%)**				
Alpha blockers	2.2	4.7*	2.2	3.1
Antiarrhythmics	7.2	9.8	7.2	8.3
Anticoagulants	3.5	3.5	3.5	3.5
Antiplatelet agents	32.3	39.7*	32.3	33.4
Beta blockers	31.4	33.7	31.4	32.1
CCB	39.3	48.3*	39.3	42.4
Digoxin	1.1	1.1	1.1	1.1
Diuretics	16.2	23.8*	16.2	17.5
Lipid modifying agents	77.9	71.6*	77.9	76.2

© 2024 Su HY et al. *JAMA Network Open*.

	Before matching		After matching	
RAAS agents	51.3	49.7	51.3	50.4
Vasodilators	17.7	20.1	17.7	19.7

Abbreviations: SGLT2is, sodium-glucose co-transporter 2 inhibitors; DPP4is, dipeptidyl peptidase 4 inhibitors; SD, standard deviation, CVD, cardiovascular disease; MI, myocardial infarction; TIA, transient ischemic attack; CHD, coronary heart disease; AMI, acute myocardial infarction; AF, atrial fibrillation; CCI, Charlson comorbidity index; TC, total cholesterol, HDL, high density lipoprotein; LDL, low density lipoprotein; HbA1c, glycated haemoglobin; aDCSI, adapted diabetes complications severity index; MPR, medication possession ratio; GLP-1RA, glucagon -like peptide-1; TZD, Thiazolidinedione, CCB, calcium channel blocker, RAAS, renin-angiotensin-aldosterone system.

Notes:

*An absolute standardized mean difference (SMD) > 0.1 indicates a significant between-group difference in baseline characteristics.

†Index date refers to the date of newly stable SGLT2i or DPP4i use in the study period.

‡CVD history was determined by patients who experienced any CVD events according to inpatient, outpatient and emergency records.

© 2024 Su HY et al. *JAMA Network Open*.

eTable 5. Results of risk of composite cardiovascular diseases associated with SGLT2i versus DPP4i use using traditional Cox proportional hazard model analyses in overall study cohort and patient subgroups

	SGLT2is		DPP4is		Hazard ratios (95% CIs) of SGLT2is versus DPP4is
	Number of events	Event rate (events/100 pys)	Number of events	Event rate (events/100 pys)	
Composite CVD (1,632 pairs of SGLT2i and DPP4i users)	316	5.303	371	5.550	0.90 (0.77, 1.05)
Individual components (1,632 pairs)					
Atrial fibrillation	39	0.588	56	0.728	0.78 (0.51, 1.17)
Coronary heart disease	235	3.803	248	3.500	1.03 (0.86, 1.23)
Heart failure	104	1.607	166	2.270	0.66 (0.51, 0.84)*
Hemorrhagic stroke	48	0.726	69	0.900	0.76 (0.52, 1.09)
Ischemic stroke	59	0.897	75	0.980	0.90 (0.63, 1.27)
Myocardial infarction	25	0.375	46	0.594	0.60 (0.37, 0.98)*
Transient ischemic attack	4	0.060	3	0.038	1.36 (0.30, 6.06)
eGFR <60mL/min/1.73m^2 (585 pairs)	159	7.902	193	8.632	0.87 (0.71, 1.08)
aDCSI score >0 (1,062 pairs)	278	7.536	323	8.826	0.85 (0.73, 1.00)
History of any CVDs (458 pairs)	212	15.984	247	18.378	0.84 (0.70, 1.01)

Abbreviations: SGLT2is, sodium-glucose co-transporter 2 inhibitors; DPP4is, dipeptidyl peptidase 4 inhibitors; CI, confidence interval; pys, person-years; CVD, cardiovascular disease; eGFR, estimated glomerular filtration rate; aDCSI, adapted diabetes complications severity index.

Notes:

✓ Composite CVD included: 1) atrial fibrillation, 2) coronary heart disease, 3) heart failure, 4) hemorrhagic stroke, 5) ischemic stroke, 6) myocardial infarction, and 7) transient ischemic attack.

✓ * indicates p-value < 0.05.

© 2024 Su HY et al. *JAMA Network Open*.

eDiscussion

Applicability of study findings to real-world settings

Against the limitations in previous studies, several methodological efforts (summarized in the table below) were undertaken in this study, which not only ensured the study robustness but also enhanced the applicability of the study findings to real-world settings.

**Limitations in previous studies**	**Methodology efforts in the present study**	**Advantages/clinical interpretations**
Traditional Cox proportional model analyses were applied, where only a single event (i.e., time-to-first event) per patient with restricted statistical assumptions (e.g., proportional hazard) was considered, thereby lacking the statistical efficiency to determine the overall benefit of SGLT2i therapy on the chronic cardiovascular burden.	The shared frailty model analysis was adopted to account for the within-individual dependence among subsequent events and unmeasured heterogeneity.	This analysis enhanced the study validity and increased the confidence of the study findings in support of real-world decision-making.
The trial data with selective populations in short-term follow-ups were utilized.	A longitudinal cohort of patients with T2D in a real-world setting was applied.	• The statistical power was enhanced by including additional events from repeat CVDs occurred over long-term follow-up.<br>• The findings enable clinicians to confirm the overall treatment effect of SGLT2is on chronic cardiovascular conditions.<br>• Long-term use of SGLT2i therapy during the progression of diabetes in the aging populations should be encouraged to lower the risk of recurrent CVDs.
Patients with high risks for CVD events were typically	Subgroup analyses for clinically	The more profound treatment effect in these

© 2024 Su HY et al. *JAMA Network Open*.

excluded from trial settings.	meaningful high-risk patients (i.e., poor renal function, multiple diabetic complications, established CVDs history) were analyzed.	vulnerable patients compared to the general T2D population highlights the urgency to optimize SGLT2i treatment to these patients in real-world settings, thereby minimizing the discrepancy between guideline recommendations and practice in the use of SGLT2is and ultimately alleviating the excess cardiovascular burden.

Abbreviations: SGLT2is, sodium-glucose co-transporter 2 inhibitors; T2D, type 2 diabetes; CVD, cardiovascular disease.

© 2024 Su HY et al. *JAMA Network Open*.
