# Supplementary material for: Cardiovascular Risks With SGLT2 Inhibitors in Clinical Practice Among Patients With Type 2 Diabetes
Source: JAMA Netw Open. 2024 Oct 30;7(10):e2441765. doi: 10.1001/jamanetworkopen.2024.41765 (PMC11525605; doi:10.1001/jamanetworkopen.2024.41765)
Supplement: Supplement 2. — Data Sharing Statement [file jamanetwopen-e2441765-s002.pdf]

Data Sharing Statement

Su. Cardiovascular Risks With SGLT2 Inhibitors in Clinical Practice Among Patients With Type 2 Diabetes. *JAMA Netw Open*. Published October 30, 2024.
doi:10.1001/jamanetworkopen.2024.41765

Data
Data available: No

Additional Information
Explanation for why data not available: Data sharing is not available in this study as data management and analyses were only allowed to be conducted in National Cheng Kung University Hospital, Taiwan owing to considerations of data privacy and safety.
